# Clinical significance of cytogenetic aberrations in bone marrow of patients with diffuse large B-cell lymphoma: prognostic significance and relevance to histologic involvement

**DOI:** 10.1186/1756-8722-6-76

**Published:** 2013-10-03

**Authors:** Seon Young Kim, Hyo Jung Kim, Hye Jin Kang, Jin Seok Kim, Hyeon Seok Eom, Tae Min Kim, Sung-Soo Yoon, Cheolwon Suh, Dong Soon Lee

**Affiliations:** 1 Department of Laboratory Medicine, Seoul National University Hospital, Seoul National University College of Medicine, 101 Daehak-ro, Jongno-gu, Seoul 110-744, Republic of Korea; 2 Department of Internal Medicine, Hallym University Sacred Heart Hospital, Hallym University College of Medicine, Anyang, Republic of Korea; 3 Department of Internal Medicine, Korea Cancer Center Hospital, Korea Institute of Radiological and Medical Sciences, Seoul, Republic of Korea; 4 Department of Internal Medicine, Severance Hospital, Yonsei University College of Medicine, Seoul, Republic of Korea; 5 Hematology-Oncology Clinic, Research Institute and Hospital, National Cancer Center, Goyang, Republic of Korea; 6 Department of Internal Medicine, Seoul National University Hospital, Seoul National University College of Medicine, Seoul, Republic of Korea; 7 Department of Internal Medicine, Asan Medical Center, University of Ulsan College of Medicine, 86 Asanbeongwon-gil, Songpa-gu, Seoul 138-736, Republic of Korea

**Keywords:** Diffuse large B-cell lymphoma, Cytogenetics, Chromosomal abnormalities, Bone marrow involvement, Prognosis

## Abstract

**Background:**

Although knowledge of the genetics of diffuse large B-cell lymphoma (DLBCL) has been increasing, little is known about the characteristics and prognostic significance of cytogenetic abnormalities and the clinical utility of cytogenetic studies performed on bone marrow (BM) specimens. To investigate the significance of isolated cytogenetic aberrations in the absence of histologic BM involvement, we assessed the implication of cytogenetic staging and prognostic stratification by a retrospective multicenter analysis of newly diagnosed DLBCL patients.

**Methods:**

We analyzed cytogenetic and clinical data from 1585 DLBCL patients whose BM aspirates had been subjected to conventional karyotyping for staging. If available, interphase fluorescence in situ hybridization (FISH) data were also collected from patients.

**Results:**

Histologic BM involvement were found in 259/1585 (16.3%) patients and chromosomal abnormalities were detected in 192 (12.1%) patients (54 patients with single abnormalities and 138 patients with 2 or more abnormalities). Isolated cytogenetic aberrations (2 or more abnormalities) without histologic involvement were found in 21 patients (1.3%). Two or more cytogenetic abnormalities were associated with inferior overall survival (OS) compared with a normal karyotype or single abnormality in both patients with histologic BM involvement (5-year OS, 16.5% vs. 52.7%; *P* < 0.001) and those without BM involvement (31.8% vs. 66.5%; *P* < 0.001). This result demonstrated that BM cytogenetic results have a significant prognostic impact that is independent of BM histology. The following abnormalities were most frequently observed: rearrangements involving 14q32, 19q13, 19p13, 1p, 3q27, and 8q24; del(6q); dup(1q); and trisomy 18. In univariate analysis, several specific abnormalities including abnormalities at 16q22-q24, 6p21-p25, 12q22-q24, and -17 were associated with poor prognosis. Multivariate analyses performed for patients who had either chromosomal abnormalities or histologic BM involvement, revealed IPI high risk, ≥ 2 cytogenetic abnormalities, and several specific chromosomal abnormalities, including abnormalities at 19p13, 12q22-q24, 8q24, and 19q13 were significantly associated with a worse prognosis.

**Conclusions:**

We suggest that isolated cytogenetic aberrations can be regarded as BM involvement and cytogenetic evaluation of BM improves staging accuracy along with prognostic information for DLBCL patients.

## Introduction

Bone marrow (BM) evaluations are an essential part of the routine staging of diffuse large B-cell lymphoma (DLBCL) [1]. DLBCL with BM involvement is rated as Ann Arbor stage IV, resulting in higher International Prognostic Index (IPI) scores and, thus, poor prognoses [2]. BM has traditionally been evaluated by morphological examination, which commonly includes immunohistochemical (IHC) staining. Histologic BM involvement has been reported in 10-30% of DLBCL cases [3,4]. Recently, additional efforts have been made to detect even a minimal involvement of lymphoma cells using flow cytometry and molecular or cytogenetic techniques. With the application of these complementary tests, approximately 10-20% of cases that were initially classified as histologically negative have been reassessed as having BM involvement [5-7]. In a previous study in which BM was evaluated using flow cytometry and immunoglobulin gene rearrangement analysis, a change in IPI was noted in 11.5% on immunophenotyping alone, and 14.1% cases on immunophenotyping and molecular testing. The revised IPI model using immunophenotyping provided better differentiation between the IPI prognostic categories [6].

Classical cytogenetic studies of BM specimens play a pivotal role in the diagnosis and prognostic prediction of many hematologic malignancies. However, the cytogenetic data concerning DLBCL tissues are limited. DLBCL is a group of B-cell malignancies that are extremely heterogeneous histopathologically, biologically, and clinically. Consistent with this heterogeneity, various chromosomal abnormalities have been reported in patients with DLBCL [8]. Correlations between cytogenetic data and clinical outcomes have been attempted for DLBCL; however, controversy remains concerning the prognostic significance of these data, most of which were obtained before the initiation of R-CHOP (rituximab, cyclophosphamide, doxorubicin, vincristine, and prednisolone) therapy.

In Korea, cytogenetic studies of BM specimens using the G-banding technique have been a routine practice in many hospitals, primarily to aid in the detection of BM involvement when staging newly diagnosed DLBCL patients. Cytogenetic study of the BM can overcome the limitations of tumor tissue cytogenetics such as fuzzy chromosomes, failure in obtaining cells in metaphase, and contamination. The presence of chromosomal aberrations in the absence of histologic involvement of BM raises the question as to whether the abnormalities truly originate from BM involving-lymphoma cells or if the aberrations are just cytogenetic noise. In the present study, to investigate the characteristics of chromosomal aberrations in the BM of DLBCL patients and to determine their prognostic significance, we retrospectively analyzed cytogenetic data of BM specimens submitted for staging from a large series of DLBCL patients.

## Materials and methods

### Study population

A total of 1585 DLBCL cases were referred from six tertiary hospitals in Korea: Seoul National University Hospital (n = 646; 1996 to 2011); Asan Medical Center (n = 484; 2001 to 2009); National Cancer Center of Korea (n = 236; 2004 to 2009); Yonsei University Hospital (n = 118; 2004 to 2009); Hallym University Hospital (n = 57; 2004 to 2009); and Korea Cancer Center Hospital (n = 44; 2005 to 2009). The cases were selected on the basis of diagnoses established according to the 2008 World Health Organization (WHO) classification criteria for primary tissue biopsy specimens [9]. BM biopsies were conducted for staging purposes at the time of the initial diagnosis. The treatment protocols were heterogeneous but generally conformed to international standards, including combination chemotherapy using CHOP-like regimens for front-line therapy, as well as salvage chemotherapy followed by stem cell transplantation for refractory cases. The baseline patient characteristics are summarized in Table 1. All of the patients were Korean, and the median age was 57 years (range, 2–91 years). A total of 1128 patients (71.2%) received R-CHOP as the initial therapy, 380 patients (24.0%) received a therapy other than R-CHOP (157 CHOP and 223 other regimens), and 77 patients (4.9%) received an unknown treatment or no treatment. The median follow-up time was 25.7 months (range, 0.1-211.1 months).This study was reviewed and approved by the institutional review board of each hospital.

**Table 1:** **The baseline characteristics of 1585 DLBCL patients and a comparison of the clinical features of the patients with histologic BM involvement (BMI**_**histo**_^**  +**^**) and those without (BMI**_**histo**_^**  –**^**)**

**Characteristics**	**Total**	**BMI**_**histo**_^** +**^	**BMI**_**histo**_^**–**^	*P *value^*^
	**(n = 1585)**	**(n = 259)**	**(n = 1326)**	
Median age, years (range)	57.4 (1.9-90.9)	59.0 (1.9-86.4)	57.0 (5.3-90.9)	0.013
Age > 60 yr	679/1585 (42.8)	124/259 (47.9)	555/1326 (41.9)	0.073
Gender (male/female, %male)	881/704 (55.6)	132/127 (51.0)	749/577 (56.5)	0.102
B symptoms	394/1585 (24.9)	135/259 (52.1)	259/1326 (19.5)	<0.001
ECOG ≥ 2	226/1585 (14.3)	86/259 (33.2)	140/1326 (10.6)	<0.001
High serum LDH	975/1585 (61.5)	203/259 (78.4)	772/1326 (58.2)	<0.001
Stage 3 or 4	822/1585 (52.6)	259/259 (100)	574/1326 (43.3)	<0.001
Stage 3 or 4, excluding BM status	809/1585 (51.0)	237/259 (91.5)	572/1326 (43.1)	<0.001
Extranodal involvement ≥ 2 sites	497/1585 (31.4)	193/259 (74.5)	304/1326 (22.9)	<0.001
International Prognostic Index				
Low risk	392/1585 (24.7)	0/259 (0)	392/1326 (29.6)	<0.001
Low/intermediate risk	462/1585 (29.2)	33/259 (12.7)	427/1326 (32.4)	
High/intermediate risk	467/1585 (29.5)	114/259 (44.0)	353/1326 (26.6)	
High risk	264/1585 (16.7)	112/259 (43.2)	152/1315 (11.5)	
Non-GCB type	500/877 (57.0)	95/150 (63.3)	405/727 (55.7)	0.086
CD5-positive	33/341 (9.7)	7/57 (12.3)	26/284 (9.2)	0.466
Initial treatment				
R-CHOP	1128/1585 (71.2)	146/259 (56.4)	982/1326 (74.1)	<0.001
CHOP	157/1585 (9.9)	30/259 (11.6)	127/1326 (9.6)	
Other treatment	223/1585 (14.1)	53/259 (20.5)	170/1326 (12.8)	
No therapy	77/1585 (4.9)	30/259 (11.6)	47/1326 (3.5)	
Initial treatment response				
CR	1020/1394 (73.2)	118/207 (57.0)	902/1187 (76.0)	<0.001
PR	268/1394 (19.2)	51/207 (24.6)	217/1187 (18.3)	
SD	33/1394 (2.4)	8/207 (3.9)	25/1187 (2.1)	
PD	73/1394 (5.2)	30/207 (14.5)	43/1187 (3.6)	
Follow-up data				
Deaths	484/1585 (30.5)	134/259 (51.7)	350/1326 (26.4)	<0.001
Median follow-up, months (range)	25.7 (0.1-211.1)	12.0 (0.1-141.4)	29.5 (0.1-211.1)	<0.001

^*^P value are based on the chi-square test for categorical variables and the Mann–Whitney *U* test for continuous variables.

The data represent the median (range) for continuous variables or the number (percentage) for categorical variables, unless otherwise indicated.

Abbreviations: BMI, bone marrow involvement; CR, complete remission; DLBCL, diffuse large B-cell lymphoma; ECOG, Eastern Cooperative Oncology Group; GCB, germinal center B-cell-like; LDH, lactic dehydrogenase; R-CHOP, rituximab, cyclophosphamide, doxorubicin, vincristine, and prednisolone; PD, progressive disease; PR, partial remission; SD, stable disease.

### Histopathology

A primary DLBCL diagnosis was established by examining hematoxylin and eosin (H&E)-stained sections of diagnostic biopsies from various tissues with IHC stains, including CD3, CD20, CD5, CD10, BCL2, BCL6, and IRF/MUM1, according to the diagnostic protocol of each institute. The type of DLBCL based on the cell of origin, either germinal center B-cell-like (GCB) or non-GCB, was defined in 877 cases using the algorithm of Hans et al. [10]. In general, the BM biopsies were performed bilaterally. Wright-stained BM smears and H&E-stained sections of BM biopsies were reviewed by hematopathologists at each institute. IHC staining was performed at the discretion of hematopathologists at each institute. The BM reports were reviewed centrally, and additional IHC staining targeting CD3, CD20, and CD79a was performed for cases with discrepant results between histologic examination and cytogenetic tests, to confirm the initial BM histologic diagnosis. The presence of benign lymphoid aggregates was distinguished from lymphoma involvement using previously described criteria [11]. Flow cytometric analysis was performed for some cases with diffuse infiltration of lymphoma cells (n = 46). According to the BM results, the cases were dichotomized into those with histological BM involvement (BMI_histo_^+^) and those without (BMI_histo_^-^).

### Cytogenetic analysis of BM

Conventional cytogenetic tests using the G-banding technique were performed on BM aspirates from all the patients. The cytogenetic tests were performed locally, and the reports were reviewed centrally by two of the investigators (SYK and DSL). Cytogenetic studies using standard techniques were performed as a part of the diagnostic work-up at the time of initial diagnosis. Conventional G-banding cytogenetic analysis was performed using the short-term unstimulated culture (24–48 h) of BM cells. At least 20 metaphases were analyzed, whenever possible. Clonal abnormalities were defined as at least two cells with the same aberration if the aberration is a chromosome gain or a structural rearrangement, or 3 or more cells with the same chromosome missing. The karyotypes were recorded according to the International System for Human Cytogenetic Nomenclature (ISCN) [12]. We classified the karyotype results according to the complexity of the chromosomal abnormalities observed. A complex karyotype was defined as ≥ 3 chromosomal abnormalities, in accordance with previous studies [13,14]. A monosomal karyotype was defined as either a single autosomal monosomy in the presence of one or more structural aberration or two or more distinct autosomal chromosome monosomies [15,16]. In the interpretation of specific abnormalities, numerical aberrations included gains, losses of chromosomes (aneuploidy) and changes in ploidy. Structural aberrations included abnormalities such as deletions, translocations, isochromosomes, and duplications. Translocations included balanced reciprocal translocations and translocations with unknown partners (additions).

Fluorescence in situ hybridization (FISH) was performed in some cases (n = 235). The following target regions were investigated using the accompanying probes: 14q32/*IGH* using an *IGH* dual-color, break-apart rearrangement probe (n = 201); *cMYC*/*IGH* using a dual-color, dual-fusion probe (n = 11); *BCL2*/*IGH* using a dual-color, dual-fusion probe (n = 29); 9p21/*p16* using a *p16*/*CEP 9* dual-color probe (n = 178); 3q27/*BCL6* using a *BCL6* dual-color, break-apart rearrangement probe (n = 95); 8q24/*cMYC* using a *cMYC* break-apart probe (n = 39) or a *cMYC*/*IGH* using dual-color, dual-fusion probe (n = 11); 1q25 using a 1p32/1q25 probe (n = 49); 17p13/*TP53* using a *P53* probe (n = 34); and 18q23/*BCL2* using a *BCL2*/*IGH* dual-color, dual-fusion probe (n = 29; all probes from Abbott/Vysis, Downers Grove, IL, USA). We analyzed interphase cells according to the manufacturer’s instruction and the ISCN criteria. At least 200 nuclei per sample were scored for normal or abnormal FISH signals. The normal cut-off values for translocation, deletion, or amplification were based on the mean (± 3SD) and the binomial distribution function [17] analyzed of 40 negative controls. The cut-off values for *IGH* break-apart probe was 2%, and *cMYC/IGH* probe was < 0.5%.

### Statistical analyses

The data were compared using the Mann–Whitney and Kruskal-Wallis tests for continuous variables and χ^2^ test for categorical variables. Each numerical abnormality and the specific locus of each structural abnormality were dichotomized as present or absent, and hierarchical clustering was performed using Pearson correlation distance metrics and Wald linkage tests. The probabilities of overall survival (OS) and progression-free survival (PFS) [18] were plotted according to the Kaplan-Meier method, and the log-rank test with Bonferroni correction for multiple testing was used to compare the survival curves. A multivariate analysis was performed using the Cox regression method. The following parameters were analyzed for multivariate analysis: advanced age, gender, IPI risk groups, history of R-CHOP treatment, BMI_histo_^+^ vs BMI_histo_^-^, ≥ 2 abnormalities vs normal karyotype or 1 abnormality, and presence of several specific cytogenetic abnormalities abnormalities, which were associated with poor prognosis in univariate analysis and found in a minimum of 5 patients. Variables in the final model were selected using stepwise selection procedure with a threshold of *P* = 0.05. The statistical analyses were performed using SPSS version 15 (SPSS, Chicago, IL, USA) and the R statistical package (R Development Team 2012). A probability level of 0.05 was considered significant in the univariate analysis. When multiple hypothesis testing was performed, the *P* value was adjusted by Bonferroni correction.

## Results

### Comparison of histology and conventional cytogenetic tests for the detection of BM involvement

A total of 259 patients (16.3%) had BM involvement, as determined through histologic examinations. Among the 259 BMI_histo_^+^ patients, 181 (69.9%) exhibited lymphoma cells on BM aspirate smears, and all 259 patients demonstrated lymphoma involvement in a BM biopsy. The median percentage of lymphoma cells in the BM aspirate smear was 6.2% (range, 0-98%). Compared with the BMI_histo_^–^ group, the BMI_histo_^+^ group had a poorer performance status, higher lactate dehydrogenase (LDH) levels, more advanced stage tumors, and more prevalent extranodal involvement; consequently, these patients had higher IPI scores (Table 1).

Chromosomal abnormalities were detected in 192/1585 patients (12.1%), of whom 124/192 (64.6%) were BMI_histo_^+^ (Table 2). Among the 192 patients with chromosomal aberrations, 42 (21.9%) exhibited the following single numerical aberrations: 33 patients had Y chromosome loss; 7 patients had a loss or gain of other single chromosomes; and 2 patients had hyperdiploid clones (Additional file 1: Table S1). Among the 42 patients with the above single numerical aberrations, 4/42 (9.5%) were BMI_histo_^+^, which was not significantly different from the proportion of BMI_histo_^+^ patients with normal karyotypes (135/1393, 9.7%; *P* = 0.971). There were 12 patients with single structural abnormalities, of whom 4 patients exhibited single deletions [del(13q) in 2 patients and del(20)(q11.2) in 2 patients], and 1 patient had a duplication of Y chromosome; none were BMI_histo_^+^. Seven patients exhibited translocations, including t(2;11)(p21;q23), t(4;10)(q28;p13), t(6;18)(p23;p11), t(10;11)(q22;q23), add(12)(q24), t(3;14)(q27;q32), and t(14;18)(q32;q21); the patients with the latter 3 types of abnormalities were BMI_histo_^+^. The remaining 138/192 patients (71.9%) exhibited ≥ 2 aberrations. Ten of these patients exhibited 2 chromosomal abnormalities: 6 patients had 1 structural aberration and 1 numerical aberration, and 4 patients had 2 structural aberrations. Among the patients with 2 chromosomal abnormalities, 6/10 (60%) were BMI_histo_^+^. A total of 128 patients exhibited complex karyotypes. Many of these patients had highly complex abnormalities, with a median of 9 total chromosomal abnormalities (range, 3–25) including many structural abnormalities (median, 7; range, 0–20). Among the patients with complex karyotypes, 111/128 were BMI_histo_^+^ (86.7%). Consequently, 117/138 patients (84.8%) with ≥ 2 chromosomal aberrations were BMI_histo_^+^. Among the patients with complex karyotypes, the percentages of abnormal metaphases were higher in the BMI_histo_^+^ group compared with the BMI_histo_^-^ group (*P* = 0.024). The percentages of metaphases with Y chromosome losses were higher in the BMI_histo_^+^ group (*P* = 0.021), however, there was no significant differences in the number of metaphases for patients with loss of single chromosomes other than Y, polyploidy or single structural abnormalities between the BMI_histo_^+^ and BMI_histo_^-^ groups. Among the patients with complex karyotypes, only a weak correlation was found between the percentage of metaphases with aberrant karyotypes and the percentage of lymphoma cells in BM aspirates (r = 0.365). Our results indicate that a finding of BM cytogenetic aberrations involving single numerical abnormalities alone cannot be regarded as sufficient evidence of BM involvement of lymphoma cells, considering the low concordance between such findings and the histologic results. If multiple chromosomal abnormalities were considered as definite evidence of the presence of lymphoma cells, the 21/1585 (1.3%) patients with isolated cytogenetic aberrations without histologic involvement would be reassessed as having BM involvement.

**Table 2:** A comparison of the histologic and conventional cytogenetic analyses

**Cytogenetic findings**	**No. of patients**	**No. of BMI**_**histo**_^**+ **^**cases**	**Percentage of abnormal metaphases**			**BM lymphoma cell percentage**^**†**^
			**BMI**_**histo**_^**+**^	**BMI**_**histo**_^**-**^	*P *value^*^	
Normal karyotype	1393 (87.9)	135/1393 (9.7)	NA	NA	NA	1 (0–98)
Abnormal karyotype	192 (12.1)	124/192 (64.6)	49 (8–100)	25 (5–100)	< 0.001	18 (0–95)
Single abnormality	54 (3.4)	7/54 (13.0)	35 (8–100)	25 (9–100)	0.697	5 (0–86)
Single numerical abnormality	42 (2.6)	4/42 (9.5)	60 (27–100)	21 (10–100)	0.038	3 (0–86)
Loss of Y	33 (2.1)	3/33 (9.1)	70 (50–100)	20 (10–100)	0.021	6 (0–86)
Loss of another single chromosome	7 (0.4)	1/7 (14.3)	27	22 (15–70)	0.617	0^‡^
Polyploidy	2 (0.1)	0/2 (0)	NA	27 (22–31)	NA	NA
Single structural abnormality	12 (0.8)	3/12 (25.0)	15 (8–35)	67 (9–100)	0.090	5 (0–29)
Multiple abnormalities	138 (8.8)	117/138 (84.8)	50 (8–100)	25 (5–100)	0.009	19 (0–95)
2 abnormalities	10 (0.7)	6/10 (60.0)	67 (10–100)	23 (15–58)	0.394	41 (12–73)
1 structural and 1 numerical abnormality	6 (0.4)	4/6 (66.7)	67 (13–92)	39 (20–58)	0.355	27 (12–50)
2 structural abnormalities	4 (0.3)	2/4 (50.0)	55 (10–100)	20 (15–25)	0.999	61 (49–73)
≥ 3 abnormalities	128 (8.1)	111/128 (86.7)	48 (8–100)	30 (5–100)	0.024	18 (0–95)
(complex karyotype)						

^*^P value by the Mann–Whitney *U* test comparing the percentage of abnormal metaphases between the BMI_histo_^+^ and BMI_histo_^-^ groups.

^†^Lymphoma cell percentage calculated from bone marrow aspirate smears.

^‡^This case showed diffuse DLBCL involvement in the bone marrow biopsy.

The data represent the median (range) for continuous variables or the number (percentage) for categorical variables, unless otherwise indicated.

*Abbreviations*: BMI_histo_^+^, histologic bone marrow involvement; BMI_histo_^-^, no evidence of histologic bone marrow involvement; NA, not applicable.

### Chromosomal abnormalities and clinical and biological characteristics

We compared the clinical and biological characteristics of the BMI_histo_^+^ patients with normal karyotypes to patients with abnormal karyotypes. Compared with the patients with normal karyotypes, the patients with a single numerical abnormality presented with less aggressive disease, as evidenced by their lower LDH levels, less involvement of extranodal sites, and lower IPI risk scores. In contrast, the patients with ≥ 2 chromosomal abnormalities had a poorer performance status, higher LDH levels, more advanced disease stages, more prevalent extranodal involvement, and thus, higher IPI scores (Table 3).

**Table 3:** **A comparison of the clinical and laboratory characteristics of patients with histologic BMI (BMI**_**histo**_^**+**^**) with normal karyotypes and patients with chromosomal abnormalities**

**Parameters**	**BMI**_**histo**_^**+ **^**and normal karyotype**	**Abnormal karyotypes**		
	**(n = 135)**	**1 numerical**	**1 structural**	**≥ 2 abnormalities**
		**(n = 42)**	**(n = 12)**	**(n = 138)**
Age (years)				
Median (range)	58 (2–86)	67 (11–83) ^*^	56 (45–77)	60 (10–86)
Age > 60 yr	64/135 (47.4)	34/42 (81.0) ^*^	5/12 (41.7)	66/138 (47.8)
Gender (male/female)	59/76 (43.7)	35/7 (83.3) ^*^	10/2 (83.3) ^*^	76/62 (55.1)
ECOG ≥ 2	26/135 (19.3)	6/42 (14.3)	0/12 (0)	62/138 (44.9) ^*^
High serum LDH	95/135 (70.4)	16/42 (38.1) ^*^	6/12 (50.0)	123/138 (89.1) ^*^
B symptoms	50/135 (37.0)	7/42 (16.7) ^*^	6/12 (50.0)	98/138 (71.0) ^*^
Stage 3 or 4,	123/135 (91.1)	18/42 (42.9) ^*^	9/12 (75.0)	126/138 (91.3)
excluding BM status				
Extranodal ≥ 2 sites	89/135 (65.9)	13/42 (31.0) ^*^	5/12 (41.7)	112/138 (81.2) ^*^
IPI risk				
Low	0/135 (0)	16/42 (38.1)	1/12 (8.3)	1/138 (0.7)
Low/intermediate	28/135 (20.7)	10/42 (23.8)	5/12 (41.7)	4/138 (2.9)
High/intermediate	65/135 (48.2)	10/42 (23.8)	6/12 (50.0)	58/138 (42.0)
High	42/135 (31.1)	6/42 (14.3) ^*^	0/12 (0) ^*^	75/138 (54.4) ^*^
Non-GCB type	46/73 (63.0)	17/26 (65.4)	3/8 (37.5)	53/88 (60.2)
CD5- positive	4/32 (12.5)	0/3 (0)	0/1 (0)	3/30 (10.0)
R-CHOP treatment	83/135 (61.5)	31/42 (73.8)	8/12 (66.7)	78/138 (56.5)
Death	53/135 (39.3)	10/42 (23.8)	2/12 (16.7)	90/138 (65.2) ^*^

^*^Significant *P* value (< 0.05) for each subgroup compared with the group of BMI_histo_^+^ patients with normal karyotypes.

The data represent the number (percentage) for categorical variables, unless otherwise indicated.

*Abbreviations*: DLBCL, diffuse large B-cell lymphoma; ECOG, Eastern Cooperative Oncology Group; GCB, germinal center B-cell-like; IPI, International Prognostic Index; LDH, lactic dehydrogenase; R-CHOP, rituximab, cyclophosphamide, doxorubicin, vincristine, and prednisolone.

### Comparison of BM histology and FISH results

The histologic examinations, conventional cytogenetics, and FISH results of a limited number of patients were compared (Table 4). Among the 235 patients for whom FISH studies were performed, there were 3 BMI_histo_^-^ patients with normal karyotypes and abnormal 14q32/*IGH* FISH results, although the frequencies of abnormal FISH signals were low (5%, 7%, and 8%). A single BMI_histo_^-^ patient exhibited cytogenetic abnormalities of the 8q24 locus despite normal FISH results using a specific probe.

**Table 4:** A comparison of conventional cytogenetic (CG) and fluorescence in situ hybridization (FISH) results

	**FISH positve**					**FISH negative**				
**Probe**	**No. of patients**	**BMI**_**histo**_^**+ **^**/**	**BMI**_**histo**_^**+ **^**/**	**BMI**_**histo**_^**- **^**/**	**BMI**_**histo**_^**- **^**/**	**No. of patients**	**BMI**_**histo**_^**+ **^**/**	**BMI**_**histo**_^**+ **^**/**	**BMI**_**histo**_^**- **^**/**	**BMI**_**histo**_^**- **^**/**
		**CG**_**whole**_^**+**^	**CG**_**whole**_^**-**^	**CG**_**whole**_^**+**^	**CG**_**whole**_^**-**^		**CG**_**locus**_^**+**^	**CG**_**locus**_^**-**^	**CG**_**locus**_^**+**^	**CG**_**locus**_^**-**^
14q32/*IGH*	37/235 (15.7)	20 (54.1)	11 (29.7)	3 (8.1)	3 (8.1)	198/235 (84.3)	6 (3.0)	54 (27.3)	0 (0.0)	138 (69.7)
9p21/p16	17/178 (9.6)	14 (82.4)	3 (17.6)	0 (0)	0 (0)	161/178 (90.4)	3 (1.9)	50 (31.1)	0 (0)	108 (67.1)
3q27/*BCL6*	19/95 (20.0)	14 (73.7)	5 (26.3)	0 (0)	0 (0)	76/95 (80.0)	2 (2.6)	28 (36.8)	0 (0)	46 (60.5)
8q24/*MYC*	7/50 (14.0)	5 (71.4)	2 (28.6)	0 (0)	0 (0)	43/50 (86.0)	3 (7.0)	12 (27.9)	1 (2.3)	27 (67.8)
1p32/1q25	9/49 (18.4)	9 (100)	0 (0)	0 (0)	0 (0)	40/49 (81.6)	1 (2.5)	21 (52.5)	0 (0)	18 (45.0)
17p13/*TP53*	3/34 (8.8)	1 (33.3)	2 (66.7)	0 (0)	0 (0)	31/34 (91.2)	0 (0)	8 (25.8)	0 (0)	23 (74.2)
18q23/*BCL2*	3/29 (10.3)	0 (0)	2 (66.7)	1 (33.3)	0 (0)	26/29 (89.7)	2 (7.7)	7 (26.9)	0 (0)	17 (65.4)

The data represent the number (percentage) for categorical variables.

*Abbreviations*: FISH, fluorescence in situ hybridization; CG_whole_^+^/CG_whole_^-^, normal or abnormal results of conventional cytogenetic tests considering whole chromosomes; CG_locus_^+^/CG_locus_^-^, normal or abnormal results of conventional cytogenetic tests for the specific locus, which is targeted by FISH probes.

### Characteristics of cytogenetic aberrations

**Figure 1:**
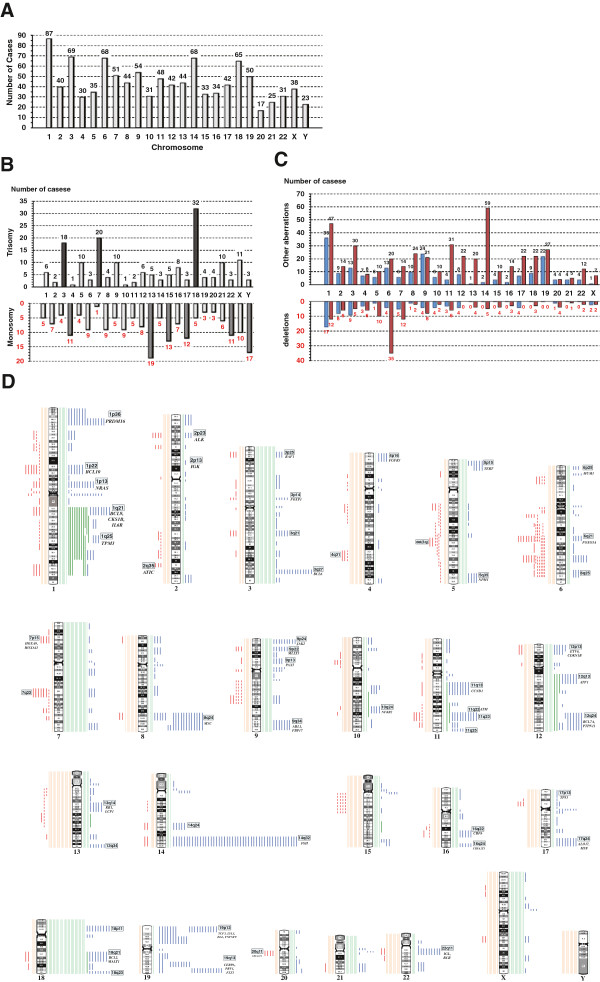
**Chromosomal aberrations in patients with chromosomal aberrations except single numerical aberrations (n = 150). (A)** The frequency of the chromosomes involved. **(B)** The frequencies of chromosomal gains (upper bars) and losses (lower bars). **(C)** The frequencies of structural aberrations in each chromosome arm (p arm, blue; q arm, red). **(D)** Ideograms showing the specific chromosomal aberrations. The orange lines on the left of the ideogram indicate chromosomal losses, and the green lines on the right side indicate gains. The red lines represent breakage points of deletions, and the blue lines indicate breakage points of chromosomal rearrangements. The thick green lines represent duplications.

We analyzed the frequencies of specific cytogenetic aberrations among the patients with chromosomal aberrations other than single numerical aberrations (n = 150) (Figure 1). The chromosomes most frequently involved were chromosome 1, 3, 6, 14, and 18. The most common numerical aberrations were trisomy 18, trisomy 7, trisomy 3, loss of Y, and loss of 13. The predominant structural aberrations involved the following loci: rearrangements involving 14q32, 19q13, 19p13, 1p32-p36, 3q27, 8q24, 18q21-q23, 1cen-1q12, 9p22-p24, 11q23-q25, 16q22-q24, and; deletions of 6q; and duplications of 1q. The well-known oncogenes and lymphoma-related genes that exhibited frequent breakpoints, including *BCL6* (3q27), *JAK2* (9p22), *IGH* (14q32), and *BCL2* (18q21), as well as other possible oncogenes with breakpoints, are indicated in Figure 1D. Monosomal karyotypes were observed in 70 patients (46.7%), and hyperdiploidy was noted in 24 cases (16.0%). Reciprocal translocations were observed in 90 patients (60.0%); among them, 19 carried reciprocal translocations involving the 14q32/*IGH* region with defined partners. The translocation partners were the following: t(8;14)(q24;q32) in 5 cases; t(14;18)(q32;q21) in 4 cases; t(3;14)(q27;q32) in 3 cases; t(1;14)(q21;q32); t(1;14)(q25;q32); t(3;14)(p25;q32); t(6;14)(q25;q32); t(9;14)(p13;q32); t(9;14)(q13;q32); and t(14;19)(q32;q13).

### Prognoses according to the chromosomal abnormalities and BM histology

**Figure 2:**
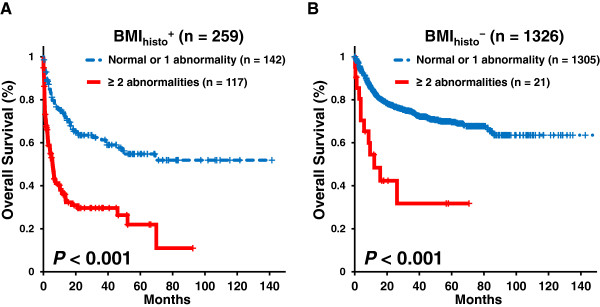
**Overall survival (OS) according to the chromosomal abnormalities and BM histology. (A)** Kaplan Meier survival curves for patients with histologic bone marrow involvement (BMI_histo_^+^; n = 259) and **(B)** for patients without histologic bone marrow involvement (BMI_histo_^-^; n = 1326). The patients with ≥ 2 chromosomal abnormalities exhibited significantly worse survival in both the BMI_histo_^+^ and BMI_histo_^-^ groups.

We analyzed the prognostic impacts of chromosomal abnormalities according to the complexity of the chromosomal aberrations involved (Figure 2). There was a significant difference in prognosis between patients with ≥ 2 abnormalities and those with a normal karyotype or a single abnormality both in the BMI_histo_^+^ group (5-year OS, 22.0% vs. 52.7%; *P* < 0.001) and BMI_histo_^-^ group (5-year OS, 31.8% vs. 66.5%; *P* < 0.001). In BMI_histo_^-^ groups, the patients with a single numerical abnormality and those with single structural abnormality did not exhibit significant differences in OS compared with those with normal karyotypes (*P* = 0.422 and 0.137, respectively). The number of BMI_histo_^+^ patients with single abnormalities was too small to assess prognostic impact (Additional file 2: Figure S1). There was no significant difference in OS and PFS according to the number of abnormalities among the patients with ≥ 2 abnormalities (Additional file 3: Figure S2).

**Figure 3:**
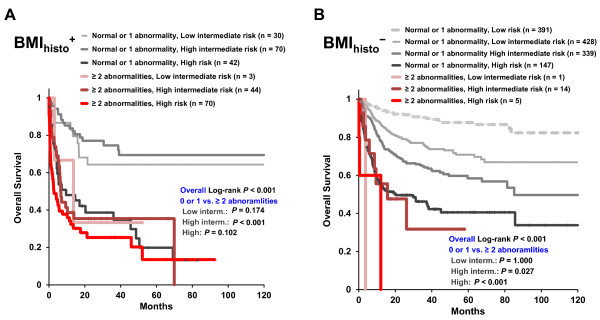
**Overall survival (OS) according to risk stratification by international prognostic index (IPI) scores and chromosomal abnormalities. (A)** Kaplan Meier survival curves for patients with histologic bone marrow involvement (BMI_histo_^+^; n = 259) and **(B)** for patients without histologic bone marrow involvement (BMI_histo_^-^; n = 1326).

When patients were classified according to IPI risk groups, BMI_histo_^+^ patients with ≥ 2 cytogenetic abnormalities presented significantly poorer prognosis compared with normal karyotypes in the high/intermediate-risk group (5-year OS, 35.4% vs. 69.4%, *P* < 0.001; Figure 3A). BMI_histo_^-^ patients with ≥ 2 abnormalities revealed significantly poorer prognosis in the high/intermediate and high risk groups (5-year OS, 31.8% vs. 58.4%, *P* = 0.027 for high/intermediate risk group; 0% vs. 40.6%, *P* < 0.001 for high risk group, respectively; Figure 3B).

When R-CHOP-treated patients were analyzed separately, ≥ 2 chromosomal abnormalities were associated with poorer prognoses compared with normal karyotypes or 1 abnormality among both the R-CHOP-treated BMI_histo_^+^ patients (5-year OS, 33.9% vs. 70.9%; *P* < 0.001) xand the BMI_histo_^-^ patients (5-year OS, 39.5% vs. 72.2%; *P* = 0.003; Additional file 4: Figure S3).

The presence of a monosomal karyotype had no apparent prognostic value among patients with ≥ 2 abnormalities (5-year OS, 25.4% vs. 20.0%; *P* = 0.274). There was no significant prognostic value of hyperdiploidy among patients with ≥ 2 abnormalities (5-year OS, 24.7% vs. 23.1%; *P* = 0.413). Among the 877 patients for whom IHC information was available, the non-GCB group demonstrated a lower OS than the GCB group (5-year OS, 58.6% vs. 71.2%, respectively; *P* = 0.004). However, among patients with ≥ 2 abnormalities, there was no significant difference in OS between the non-GCB and GCB types (5-year OS, 25.4% vs. 23.4%, respectively; *P* = 0.467).

### Prognoses according to specific chromosomal abnormalities

**Figure 4:**
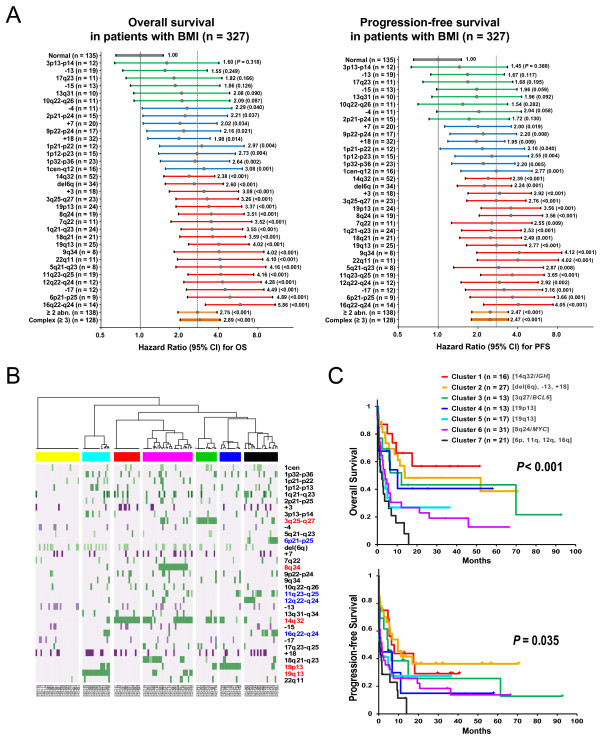
**Prognoses according to specific chromosomal abnormalities. (A)** Forest plots showing hazard ratios obtained by univariate Cox analysis for overall survival (OS) and progression-free survival (PFS) according to the presence of specific chromosomal abnormalities using patients with lymphoma bone marrow involvement and normal karyotype as a reference group in 327 patients having either abnormal karyotypes or histologic BM involvement. **(B)** Based on a cluster analysis of 33 frequent numerical abnormalities and breakpoints, 138 patients with ≥ 2 chromosomal abnormalities were segregated into clusters with characteristic abnormality patterns. The horizontal labels indicate clusters (red, Cluster 1; yellow, Cluster 2; green, Cluster 3; blue, Cluster 4; cyan, Cluster 5; purple, Cluster 6; and black, Cluster 7). The characteristic loci of the abnormalities in each cluster are indicated in red, and the loci associated with a poor prognosis in the univariate analysis are indicated in blue in the right panel. **(C)** The (OS) and PFS of each cluster is plotted.

We investigated the prognostic impacts of the specific chromosomal abnormalities. Figure 4A presents hazard ratios (HRs) obtained by univariate Cox analysis for OS and PFS according to specific chromosomal abnormalities found in ≥ 8 patients using BMI_histo_^+^ patients with normal karyotype as a reference group among 327 patients with either cytogenetic abnormalities or were BMI_histo_^+^. The presence of structural abnormalities at 16q22-q24 was significantly associated with a higher risk for both OS (HR, 5.86, *P* < 0.001) and disease progression (HR, 4.05, *P* < 0.001). The abnormalities at 6p21 (HR for OS, 4.89, *P* < 0.001), 12q22-q24 (HR, 4.28, *P* < 0.001), and -17 (HR, 4.49, *P* < 0.001) were also significant association with adverse prognosis. Consistent results were observed in the R-CHOP-treated patients (Additional file 5: Figure S4). In addition, frequent loci of cytogenetic abnormalities, including 11q21-q23, 19q13, 18q21, 1q21-q23, 8q24, 19p13, 3q27, 6q, and 14q32 were also associated with adverse outcomes.

To identify the subgroups of BM-involved DLBCL that exhibited distinct cytogenetic aberrations, we performed cluster analyses using the loci associated with poor survival in the univariate analysis and frequent and characteristic breakpoints, including 14q32, 3q27, 8q24, 19p13, and 19p13. Based on the hierarchical cluster analysis, 138 patients with ≥ 2 chromosomal abnormalities were segregated into clusters with characteristic patterns of chromosomal abnormalities (Figure 4B). When we compared prognoses among the clusters, Cluster 7, which was composed of loci associated with poorer prognoses in the univariate analysis, revealed the poorest prognosis (Figure 4C).

### Multivariate analysis of prognoses among patients with BM abnormalities diagnosed by either histologic examination or conventional cytogenetic testing

A multivariate analysis was performed for 327 patients who had either chromosomal abnormalities or were BMI_histo_^+^ (Table 5). When the presence of ≥ 2 cytogenetic abnormalities was analyzed with other covariates, the presence of ≥ 2 cytogenetic abnormalities was also significantly associated with a worse OS (HR, 2.49; 95% CI, 1.75-3.54; *P* < 0.001). The high IPI score was strongly associated with a poor prognosis, whereas R-CHOP treatment was strongly associated with a better prognosis. When the specific chromosomal abnormalities which were associated with adverse prognosis in the univariate analysis were analyzed, the aberration at 19p13 was selected as an independent adverse prognostic factor (HR, 2.67; 95% CI, 1.50-4.76; *P* = 0.001), in addition to 7q22, 12q22-q24, 18q21, and 16q22-q24. When PFS was analyzed, aberrations at 19p13 and 8q24 emerged as factors independently associated with disease progression (HR, 3.02 and 2.61, respectively; *P* < 0.001 and *P* < 0.001, respectively). When 200 R-CHOP treated patients among 327 patients were analyzed separately, 19q13 (HR, 3.36, *P* = 0.003), 12q22-q24, 19p13, and 8q24 were independently predicted poor OS, and 19p13 and 8q24 were associated with disease progression (Table 5).

**Table 5:** **Multivariate Cox analysis of the overall survival (OS) and progression-free survival (PFS) among 327 patients having either cytogenetic abnormalities (CAs) or histologic BM involvement (BMI**_**histo**_^**+**^) and among R-CHOP treated patients (n = 200)^*^

		**OS**			**PFS**		
**Risk factors**	**No. of patients (%)**	**HR**	**95% CI**	***P***	**HR**	**95% CI**	***P***
**Total patients with CAs and/or BMI**_**histo**_^**+ **^**(n = 327)**							
Presence of ≥ 2 cytogenetic abnormalities as a risk factor							
IPI risk group, high vs. low	123 (37.6)	6.46	1.56-26.72	0.010	7.15	1.73-29.46	0.007
R-CHOP treatment, R-CHOP vs. others	200 (61.2)	0.26	0.18-0.35	<0.001	0.38	0.28-0.51	<0.001
≥ 2 cytogenetic abnormalities vs. 0 or 1 abnormality	138 (42.2)	2.49	1.75-3.54	<0.001	2.12	1.53-2.93	<0.001
Presence of specific cytogenetic abnormalities as risk factors							
IPI risk group, high vs. low	123 (37.6)	8.34	2.04-24.09	0.003	8.43	2.06-34.44	0.003
R-CHOP treatment, R-CHOP vs. others	200 (61.2)	0.24	0.17-0.34	<0.001	0.32	0.24-0.44	<0.001
19p13 abnormality present vs. absent	24 (7.3)	2.67	1.50-4.76	0.001	3.02	1.85-4.93	<0.001
7q22 abnormality present vs. absent	11 (3.4)	3.03	1.51.-6.06	0.002	NS	NS	NS
12q22-q24 abnormality present vs. absent	12 (3.7)	2.68	1.37.-5.26	0.004	NS	NS	NS
18q21 abnormality present vs. absent	21 (6.4)	2.11	1.22.-3.64	0.007	NS	NS	NS
16q22-q24 abnormality present vs. absent	14 (4.3)	2.27	1.23-4.18	0.009	NS	NS	NS
11q23-q25 abnormality present vs. absent	19 (5.8)	1.81	1.03-3.15	0.038	NS	NS	NS
8q24 abnormality present vs. absent	19 (5.8)	NS	NS	NS	2.61	1.54-4.43	<0.001
**R-CHOP treated patients with CAs and/or BMI**_**histo**_^**+ **^**(n = 200)**							
Presence of ≥ 2 cytogenetic abnormalities as a risk factor							
IPI risk group, high vs. low	67 (33.5)	5.29	1.23-22.86	0.026	5.55	1.32-23.37	0.020
Male vs. female	112 (56.0)	1.71	1.01-2.91	0.046	NS	NS	NS
≥ 2 cytogenetic abnormalities vs. 0 or 1 abnormality	78 (39.0)	2.01	1.17-3.45	0.012	2.15	1.38-3.38	0.001
Presence of specific cytogenetic abnormalities as risk factors							
IPI risk group, high vs. low	67 (33.5)	5.92	1.40-25.04	0.016	7.61	1.82-31.92	0.006
Male vs. female	112 (56.0)	2.00	1.15-3.47	0.014	1.71	1.09-2.69	0.021
19q13 abnormality present vs. absent	13 (6.5)	3.36	1.50-7.54	0.003	NS	NS	NS
12q22-q24 abnormality present vs. absent	6 (3.0)	3.65	1.42-9.44	0.007	NS	NS	NS
19p13 abnormality present vs. absent	16 (8.0)	2.85	1.20-6.80	0.018	3.31	1.68-6.51	0.001
8q24 abnormality present vs. absent	13 (6.5)	2.54	1.24-5.23	0.011	2.43	1.24-4.73	0.009

*The multivariate Cox regression models initially included age, gender, the International Prognostic Index (IPI) risk groups, history of R-CHOP treatment, presence of histologic bone marrow involvement, and either presence of ≥ 2 cytogenetic abnormalities, or presence of specific cytogenetic abnormalities found in minimum 5 R-CHOP-treated patients and associated with significant poor prosnosis in the univariate analysis (+3, -17, abnormalities at 14q32, 3q25-q27, 19p13, 8q24, 7q22, 1q21-q23, 18q21, 19q13, 9q34, 22q11, 11q23-q25, 12q22-q24, 16q22-q24, and deletion 6q). The stepwise selection procedure was used to select variables in the final models.

Abbreviations: CI, confidence interval; HR, hazard ratio; NS, not significant.

## Discussion

With recent advances in genetic technology, the value of G-banding data is often underestimated compared with the data derived from higher-resolution techniques, such as FISH, array-CGH, and deep sequencing. Molecular testing for immunoglobulin gene rearrangements is also a sensitive test for detecting clonal cells within bone marrow, which detected BM positive cases in 16% of histologically negative cases in a previous study [6,19]. Although conventional karyotyping has low resolution, this method remains the only technique used worldwide in many hospital laboratories; thus, it is readily applicable in routine practice. In addition, the advantages of conventional cytogenetic tests include their ability to detect abnormalities in proliferative clones; to provide information regarding whole chromosomes, including balanced translocations; and to distinguish between heterogeneous clones that coexist in a sample.

In fact, conventional cytogenetic testing is not as widely performed for DLBCL as for other hematologic malignancies, such as acute leukemia and multiple myeloma. The disadvantages of cytogenetic testing using tissue samples include the necessity of laborious tissue preparation, the presence of fuzzy chromosomes, a lack of metaphase cells, and contamination. Because BM studies are a standard process of DLBCL staging, the use of BM samples for additional conventional cytogenetic testing is easily applicable. There are two major possible advantages associated with the cytogenetic testing of BM samples in DLBCL patients: an increased sensitivity for detecting BM involvement in DLBCL and the information provided by the chromosomal abnormalities found in the BM. In this study, we analyzed retrospective data from a large series of patients to analyze both possible advantages.

One major difficulty in the decision about whether to perform cytogenetic testing on BM specimens is caused by the absence of BM involvement in many DLBCL patients. Even in patients with BM involvement, the lymphoma cells may be obscured by the more abundant normal hematopoietic cells [5,6,19,20]. In our data, 8.8% of the total patients exhibited multiple chromosomal abnormalities, and 1.3% of the patients were diagnosed with BM involvement on the basis of cytogenetic results alone. Considering that 16.3% of the cases in our study were BMI_histo_^+^, the detection rate of significant cytogenetic abnormalities in BM specimens was not negligible. Because karyotyping is based on metaphases, the number of metaphases with cytogenetic abnormalities does not necessarily represent the proportion of cells within BM cells [21]. Karyotyping may be a sensitive method, because a small clone with proliferative advantage over normal cells may be identified by karyotyping.

The interpretation of data from patients with a single abnormality can be difficult because the abnormality does not necessarily originate from lymphoma cells. In our study, single aneuploidies demonstrated only a 10% concordance rate with histologic examination, which indicates that a single aneuploidy cannot be the definite evidence for presence of lymphoma cells. In addition, patients with single aneuploidies had less aggressive disease than normal karyotype cases with histologic involvement and presented no significant differences in prognosis. Therefore, the presence of a single numerical abnormality cannot be an indicator of the advanced disease and a poor prognostic factor. In fact, loss of chromosome Y is a well-known normal age-related phenomenon in elderly males [22]. Although monosomy 21 can be detected as a sole cytogenetic abnormality in a variety of hematologic disorders, the random loss of chromosome as an artifact of cell culture or slide preparation can cause this abnormality [23]. Single structural abnormalities also presented lower concordance rate with histologic BM involvement. There were 2 patients with del(20q) without histologic evidence of BM involvement. Although these patients did not presented cytopenia or morphologic dysplasia, previous studies reported that myelodysplastic syndrome associated with isolated del(20q) can commonly present with minimal morphological dysplasia [24]. There were a patient with duplication of Y chromosome and patients with balanced translocation, which may be constitutional chromosomal aberrations [25,26]. However, some single abnormalities can represent a primary event in lymphomagenesis. In this study, single structural abnormalities such as t(14;18)(q32;q21) or t(3;14)(q27;q32) can be reasonably regarded as originated from lymphoma cells considering their well-known chromosomal loci and their concordant histologic BM results. Because of the small number of analyzed cases, it was hard to make convincing conclusion about the significance and prognostic impact of these single abnormalities in this study. When these abnormalities are detected, cautious BM examination with other ancillary tests may be needed. Further studies will be needed for more clear conclusion and to make a guideline on the appropriate interpretation of ambiguous cytogenetic abnormalities.

Most importantly, this study demonstrated that BM cytogenetic results have a powerful prognostic significance that is independent of BM histology. The presence of ≥ 2 chromosomal abnormalities was associated with very poor prognosis among both patients with and without histologic BM involvement. This finding confirms that a BM cytogenetic result is a stronger prognostic predictor than histologic BM involvement. The significant prognostic effect of cytogenetic abnormalities was observed in patients that had been stratified according to their IPI risk scores, especially for patients with high intermediate risks.

The chromosomal abnormalities observed in this study were nonrandom and recurrent, implying that they may belong to the recurrent lymphomagenesis pathway [27]. There have been previous reports of high frequencies of many of these abnormalities, including rearrangements at 14q32, 1p, 3q27, 8q24, 11q23-q25, and 18q21-q23; duplications at 1q; and deletions at 6q. The most common numerical abnormality in DLBCL, trisomy 18, has also been frequently reported in many other B-cell neoplasms [28,29]. The 14q32/*IGH* rearrangement, which was the single most common abnormality in the present study, is frequently observed in all B-cell neoplasia [30-36]. Because 14q32/*IGH* rearrangements mainly involve balanced translocations, these rearrangements can be detected only by G-banding, not through array methods [37-41].

In our data from patients with multiple abnormalities, which specific loci presented abnormalities was a more significant prognostic factor than the number of abnormalities. Several abnormalities, including aberrations at 16q22-q24, 6p21-p25, 12q22-q24, 11q23-q25, 19q13, 1q21-q23, 8q24, and 19p13, and -17 appeared to be associated with a worse prognosis in the univariate analysis. The multivariate analysis demonstrated that several chromosomal abnormalities including aberrations at 19p13, 7q22, 12q22-q24, 18q21, and 16q22-q24 are independent adverse prognostic factors for survival among DLBCL patients with BM involvement. In addition, abnormalities at 19q13, 12q22-q24, 19p13, and 8q24 were associated with poor prognoses among R-CHOP treated patients with BM abnormalities. Because of the small number of patients having each specific cytogenetic abnormality, and the high variability of the cytogenetic abnormalities, to make clear conclusion about the prognostic importance of each specific chromosomal loci might be difficult. However, we considered a recurrent cytogenetic abnormality with a high statistical significance, such as abnormalities at 19p13 can be a potentially important target for further investigation. Aberrations in chromosome 19 have been reported in several previous series of DLBCL cases, although their prognostic impact was not explored [30,33,35,36,40-42]. The identity of the specific gene on 19p13 that is associated with DLBCL is not yet known; however, *TCF3* has been reported to be associated with acute lymphoblastic leukemia [43], and microRNA (miRNA) genes have been implicated in mature B-cell neoplasia [44]. In addition, a recent study using array-CGH and SNP-chip analyses reported that recurrent deletions of the tumor suppressor genes, *TNFSF7* and *TNFSF9* at the 19p13.3 region were observed in DLBCL and Burkitt lymphomas [45]. These genes may play an important role in the pathogenesis of DLBCL leading to disease progression and BM involvement. The *MYC*/8q24 rearrangement, which has been reported to be associated with a poor prognosis when detected in tissues using FISH at the time of diagnosis [46-49], was independently associated with disease progression in this study. The nonrandom nature of the observed chromosomal abnormalities and their association with prognosis suggest that investigations into the clonal evolution of DLBCL could provide useful insights into the pathogenesis of this disease.

Our study has several limitations. First, this study was performed retrospectively, and the patients had undergone heterogeneous therapies. Second, the evaluations of the cell of origin and CD5 positivity were limited. Third, several BM-related prognostic factors, including the extent and histologic characteristics of the BM involvement, could not be investigated [3,4,50,51]. However, considering the comparable results of the BMI_histo_^+^ and BMI_histo_^-^ cases, it could be suggested that cytogenetic aberrations are a stronger prognostic factor than other BM-related factors.

The results of this study may be representative of the major characteristics of the BM chromosomal abnormalities in the Korean DLBCL population, considering the large size of our data series. A larger series of international investigations may be needed to characterize and confirm the prognostic significance of the BM cytogenetic aberrations in DLBCL patients from other ethnic groups, considering the variety of external genotoxic agents that can cause chromosomal changes and the diversity of host susceptibility factors to chromosomal breaks. Results from classic cytogenetics can guide the design of additional studies using other techniques, such as FISH or arrays.

In conclusion, the conventional cytogenetic testing of BM may provide essential information for newly diagnosed DLBCL patients. We suggest that cytogenetic testing of BM samples should be integrated with DLBCL staging apart from histologic examination, and more effective therapeutic strategies should be developed. In addition, frequently affected cytogenetic regions, such as 19p13, must be intensively investigated to characterize the underlying molecular pathogenesis of DLBCL.

## Competing interests

The authors declare that they have no competing interests.

## Authors’ contributions

CS and DSL designed the study, SYK, HJK, HJK, JSK, HSE, TMK, SSY, CS, and DSL provided study materials or patients, collected and assembled data. SYK, CS, and DSL analyzed and interpreted data. SYK, HJK, HJK, JSK, HSE, TMK, SSY, CS, and DSL wrote the manuscript. All authors have read and approved the final manuscript.

## Supplementary Material

Additional file 1: Table S1 Detailed karyotypes of 192 patients with chromosomal abnormalities. Karyotype results were classified according to the complexity of chromosomal abnormalities observed. Click here for file

Additional file 2: Figure S1 Kaplan-Meier survival plots of OS according to histologic bone marrow involvement (BMI) and chromosomal abnormalities found in bone marrow cells in DLBCL patients. **(A)** The patients with a single abnormality did not exhibit significant differences in OS in patients without bone marrow involvement (BMI_histo_^-^). **(B)** The patients with complex karyotypes (≥3 abnormaliteis) exhibited significantly worse survival in both the BMI_histo_^+^ and BMI_histo_^-^ groups. Click here for file

Additional file 3: Figure S2 Survival according to the total number of chromosomal abnormalities in 1585 DLBCL patients. (**A)** overall survival (OS). **(B)** progression-free survival (PFS). The patients with ≥ 2 abnormalities exhibited significantly worse OS and PFS; however, there was no significant difference according to the number of abnormalities among patients with ≥ 2 abnormalities. Click here for file

Additional file 4: Figure S3 Kaplan-Meier survival plots of overall survival (OS) of R-CHOP treated DLBCL patients according to chromosomal abnormalities **(A)** in patients with histologic bone marrow involvement (BMI_histo_^+^) and **(B)** in patients without histologic bone marrow involvement (BMI_histo_^-^). **(C and D)** OS according to risk stratification by international prognostic index (IPI) scores and chromosomal abnormalities **(C)** in R-CHOP-treated BMI_histo_^+^ patients and **(D)** in R-CHOP-treated BMI_histo_^-^ patients. Click here for file

Additional file 5: Figure S4 Forest plots showing hazard ratios (HRs) obtained by univariate Cox analysis for **(A)** overall survival (OS) and **(B)** progression-free survival (PFS) according to the presence of specific chromosomal abnormalities using patients with lymphoma bone marrow involvement and normal karyotype as a reference group in R-CHOP-treated patients having either abnormal karyotypes or histologic BM involvement (n = 200). Click here for file
