# Supplementary material for: Clinical significance of cytogenetic aberrations in bone marrow of patients with diffuse large B-cell lymphoma: prognostic significance and relevance to histologic involvement
Source: J Hematol Oncol. 2013 Oct 3;6:76. doi: 10.1186/1756-8722-6-76 (PMC3851800; doi:10.1186/1756-8722-6-76)
Supplement: Additional file 2: Figure S1 — Kaplan-Meier survival plots of OS according to histologic bone marrow involvement (BMI) and chromosomal abnormalities found in bone marrow cells in DLBCL patients. (A) The patients with a single abnormality did not exhibit significant differences in OS in patients without bone marrow involvement (BMIhisto-). (B) The patients with complex karyotypes (≥3 abnormaliteis) exhibited significantly worse survival in both the BMIhisto+ and BMIhisto- groups. [file 1756-8722-6-76-S2.pdf]

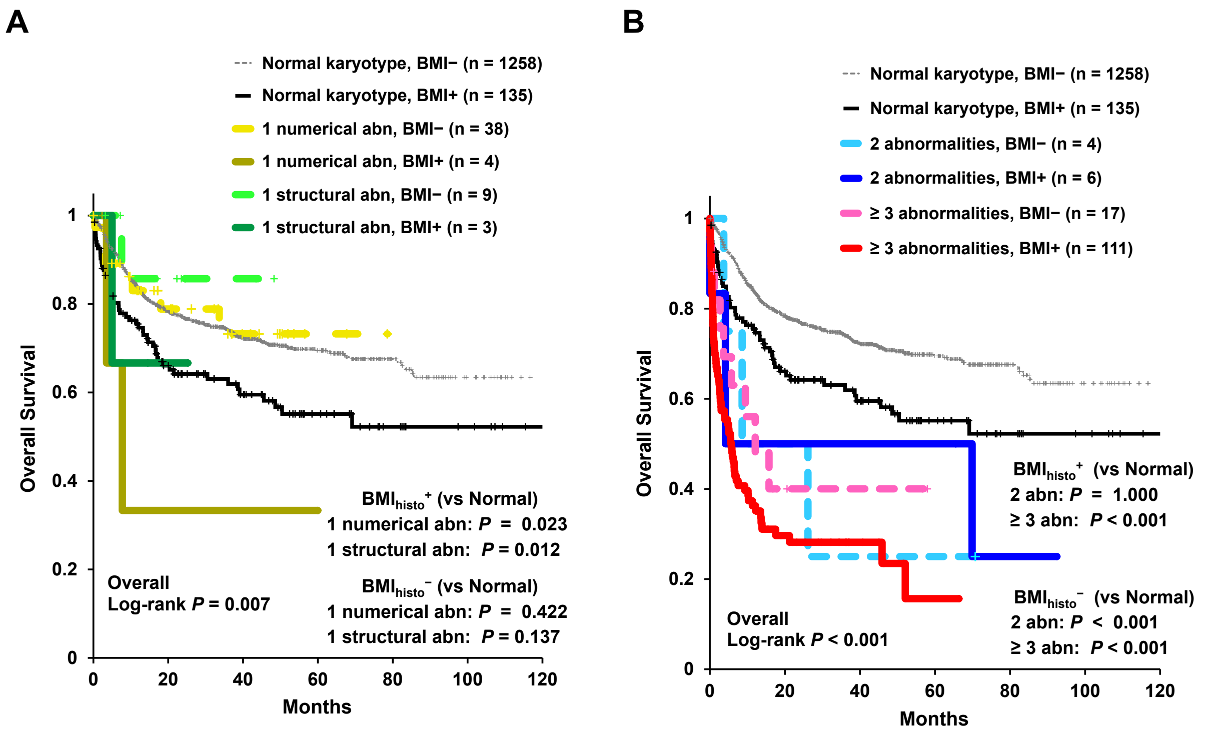

Supplementary Figure S1. Kaplan-Meier survival plots of OS according to histologic bone marrow involvement (BMI) and chromosomal abnormalities found in bone marrow cells in DLBCL patients. **(A)** The patients with a single abnormality did not exhibit significant differences in OS in patients without bone marrow involvement (BMI$_{histo}^{-}$). **(B)** The patients with complex karyotypes (≥3 abnormaliteis) exhibited significantly worse survival in both the BMI$_{histo}^{+}$ and BMI$_{histo}^{-}$ groups.
