# Supplementary material for: Clinical significance of cytogenetic aberrations in bone marrow of patients with diffuse large B-cell lymphoma: prognostic significance and relevance to histologic involvement
Source: J Hematol Oncol. 2013 Oct 3;6:76. doi: 10.1186/1756-8722-6-76 (PMC3851800; doi:10.1186/1756-8722-6-76)
Supplement: Additional file 3: Figure S2 — Survival according to the total number of chromosomal abnormalities in 1585 DLBCL patients. (A) overall survival (OS). (B) progression-free survival (PFS). The patients with ≥ 2 abnormalities exhibited significantly worse OS and PFS; however, there was no significant difference according to the number of abnormalities among patients with ≥ 2 abnormalities. [file 1756-8722-6-76-S3.pdf]

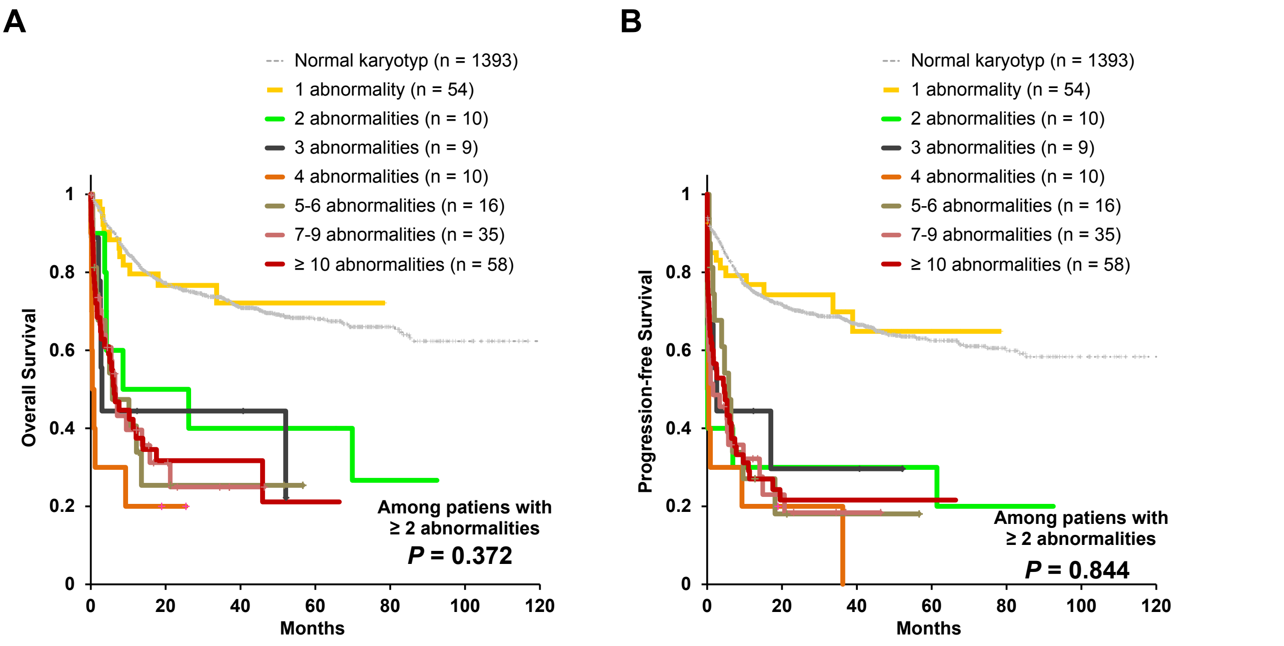

Supplementary Figure S2. Survival according to the total number of chromosomal abnormalities in 1585 DLBCL patients. (**A)** overall survival (OS). (**B)** progression-free survival (PFS). The patients with ≥ 2 abnormalities exhibited significantly worse OS and PFS; however, there was no significant difference according to the number of abnormalities among patients with ≥ 2 abnormalities.
