# Supplementary material for: Clinical significance of cytogenetic aberrations in bone marrow of patients with diffuse large B-cell lymphoma: prognostic significance and relevance to histologic involvement
Source: J Hematol Oncol. 2013 Oct 3;6:76. doi: 10.1186/1756-8722-6-76 (PMC3851800; doi:10.1186/1756-8722-6-76)
Supplement: Additional file 4: Figure S3 — Kaplan-Meier survival plots of overall survival (OS) of R-CHOP treated DLBCL patients according to chromosomal abnormalities (A) in patients with histologic bone marrow involvement (BMIhisto+) and (B) in patients without histologic bone marrow involvement (BMIhisto-). (C and D) OS according to risk stratification by international prognostic index (IPI) scores and chromosomal abnormalities (C) in R-CHOP-treated BMIhisto+ patients and (D) in R-CHOP-treated BMIhisto- patients. [file 1756-8722-6-76-S4.pdf]

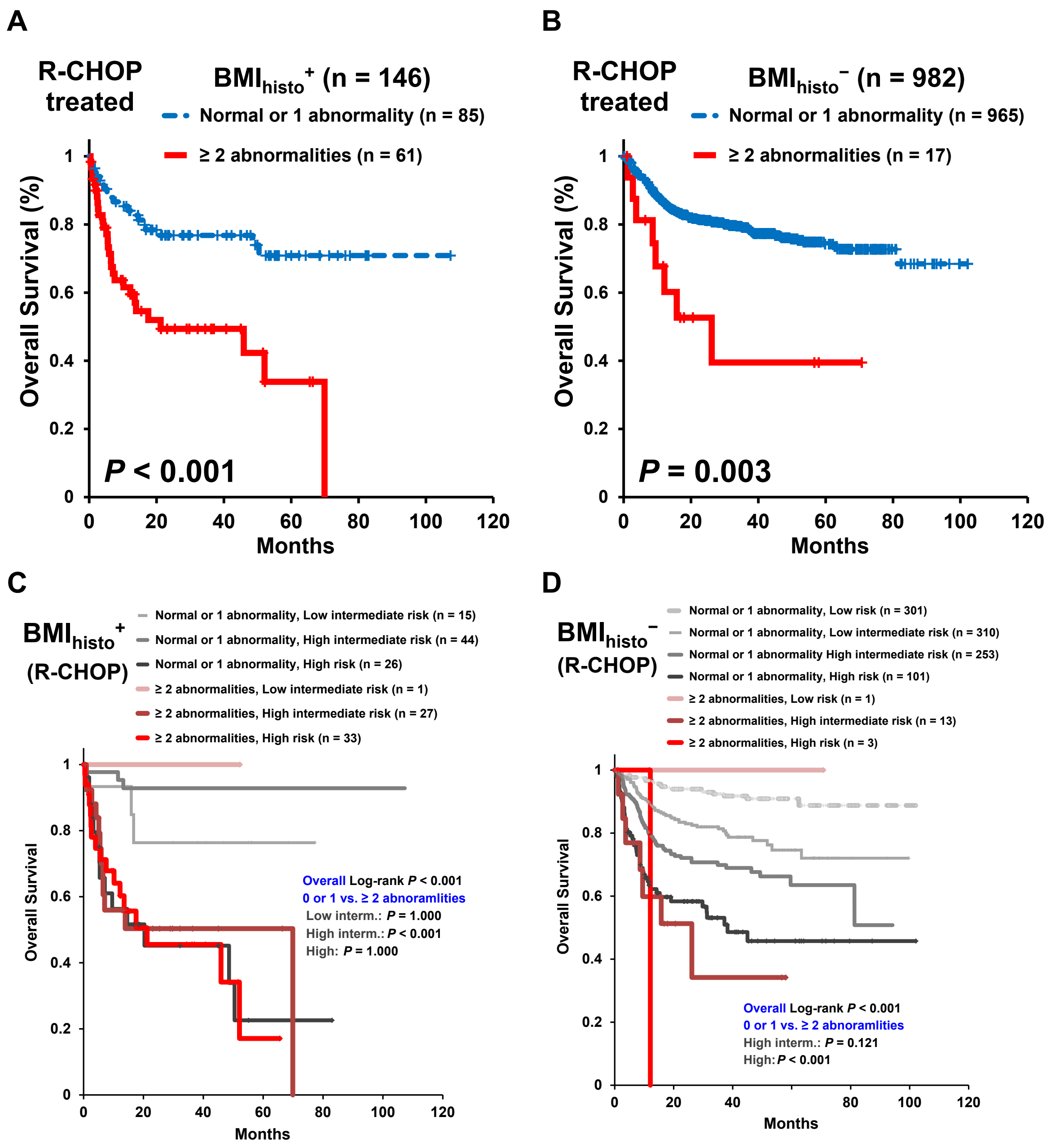

Supplementary Figure S3. Kaplan-Meier survival plots of overall survival (OS) of R-CHOP treated DLBCL patients according to chromosomal abnormalities **(A)** in patients with histologic bone marrow involvement (BMI_{histo}^{+}) and **(B)** in patients without histologic bone marrow involvement (BMI_{histo}^{-}). **(C and D)** OS according to risk stratification by international prognostic index scores and chromosomal abnormalities **(C)** in R-CHOP-treated BMI_{histo}^{+} patients and **(D)** in R-CHOP-treated BMI_{histo}^{-} patients.
