# Supplementary material for: Clinical significance of cytogenetic aberrations in bone marrow of patients with diffuse large B-cell lymphoma: prognostic significance and relevance to histologic involvement
Source: J Hematol Oncol. 2013 Oct 3;6:76. doi: 10.1186/1756-8722-6-76 (PMC3851800; doi:10.1186/1756-8722-6-76)
Supplement: Additional file 5: Figure S4 — Forest plots showing hazard ratios (HRs) obtained by univariate Cox analysis for (A) overall survival (OS) and (B) progression-free survival (PFS) according to the presence of specific chromosomal abnormalities using patients with lymphoma bone marrow involvement and normal karyotype as a reference group in R-CHOP-treated patients having either abnormal karyotypes or histologic BM involvement (n = 200). [file 1756-8722-6-76-S5.pdf]

A B

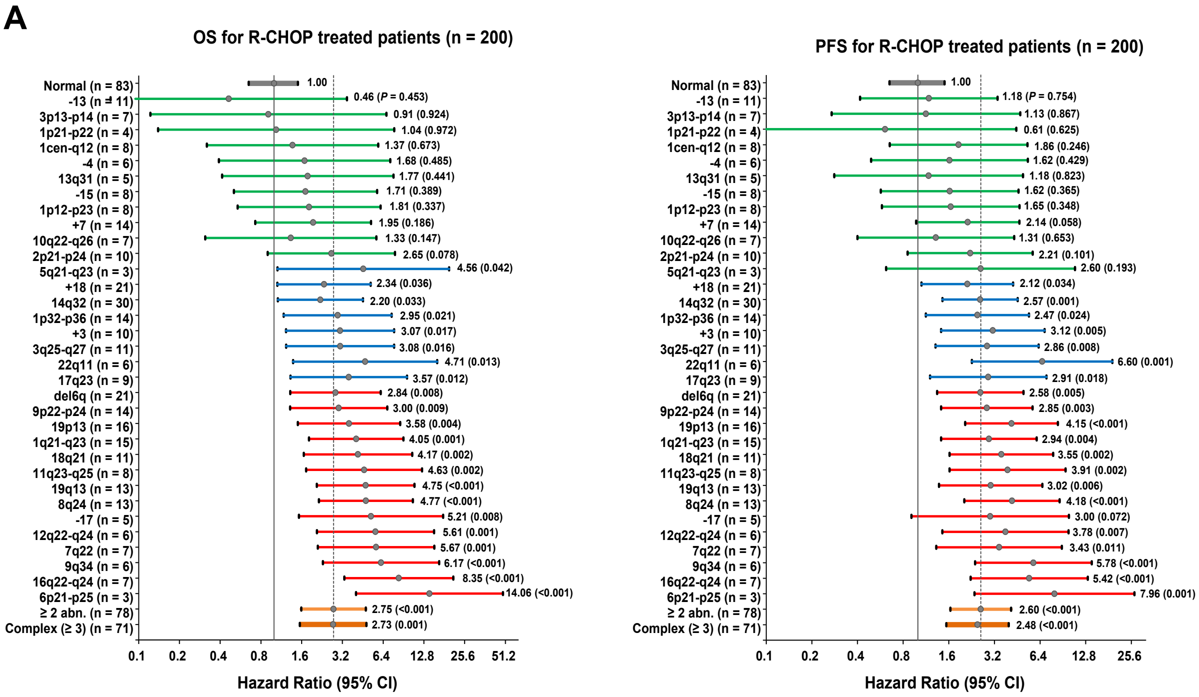


**Supplementary Figure S4.** Forest plots showing hazard ratios (HRs) obtained by univariate Cox analysis for **(A)** overall survival (OS) and **(B)** progression-free survival (PFS) according to the presence of specific chromosomal abnormalities using patients with lymphoma bone marrow involvement and normal karyotype as a reference group in R-CHOP-treated patients having either abnormal karyotypes or histologic BM involvement (n = 200).
